# On-Site Detection of Carcinoembryonic Antigen in Human Serum

**DOI:** 10.3390/bios11100392

**Published:** 2021-10-14

**Authors:** Tohid Mahmoudi, Mohammad Pourhassan-Moghaddam, Behnaz Shirdel, Behzad Baradaran, Eden Morales-Narváez, Hamed Golmohammadi

**Affiliations:** 1 Immunology Research Center, Tabriz University of Medical Sciences, Tabriz 5166-15731, Iran; mahmoodit@tbzmed.ac.ir (T.M.); behnaz.shirdel201467@gmail.com (B.S.); baradaranb@tbzmed.ac.ir (B.B.); 2 School of Life Sciences, Faculty of Science, University of Technology Sydney, Sydney, NSW 2007, Australia; m.pourhassan_moghaddam@unsw.edu.au; 3 ARC Research Hub for Integrated Device for End-User Analysis at Low-Levels (IDEAL Research Hub), Faculty of Science, University of Technology Sydney, Sydney, NSW 2007, Australia; 4 Biophotonic Nanosensors Laboratory, Centro de Investigaciones en Óptica, A. C. Loma del Bosque 115, Lomas del Campestre, León 37150, Guanajuato, Mexico; 5 Nanosensor Bioplatforms Laboratory, Chemistry and Chemical Engineering Research Center of Iran, Tehran 1496-813151, Iran

**Keywords:** lateral flow immunoassay, carcinoembryonic antigen, cancer diagnosis, smartphone-based sensors, point-of-care testing

## Abstract

Real-time connectivity and employment of sustainable materials empowers point-of-care diagnostics with the capability to send clinically relevant data to health care providers even in low-resource settings. In this study, we developed an advantageous kit for the on-site detection of carcinoembryonic antigen (CEA) in human serum. CEA sensing was performed using cellulose-based lateral flow strips, and colorimetric signals were read, processed, and measured using a smartphone-based system. The corresponding immunoreaction was reported by polydopamine-modified gold nanoparticles in order to boost the signal intensity and improve the surface blocking and signal-to-noise relationship, thereby enhancing detection sensitivity when compared with bare gold nanoparticles (up to 20-fold in terms of visual limit of detection). Such lateral flow strips showed a linear range from 0.05 to 50 ng/mL, with a visual limit of detection of 0.05 ng/mL and an assay time of 15 min. Twenty-six clinical samples were also tested using the proposed kit and compared with the gold standard of immunoassays (enzyme linked immunosorbent assay), demonstrating an excellent correlation (R = 0.99). This approach can potentially be utilized for the monitoring of cancer treatment, particularly at locations far from centralized laboratory facilities.

## 1. Introduction

Cancer is the main cause of morbidity and mortality around the world, with an approximated 18.1 million recent patients and 9.6 million deaths in 2018 [1]. Despite the significant progress in cancer treatment in recent years, the current methodologies have still failed to reach completely satisfactory results, mainly due to late detection. Hence, the early diagnosis of cancer via the quantification of some biomarkers can be considered the golden step for its timely treatment, since it increases the successful treatment rate and, consequently, reduces the related costs and health burden. Moreover, the monitoring of cancer biomarkers is necessary during the treatment process, which further intensifies the importance of the development of on-site diagnostic devices [2].

The carcinoembryonic antigen (CEA) is a collection of glycoproteins that are usually produced for the duration of fetal development, but its production ends prior to birth. Its increase is mostly utilized as a tumor marker to monitor the treatment of colorectal carcinoma or other carcinomas, to recognize recurrences, and for the staging of tumors [3]. Enzyme-linked immunosorbent assay (ELISA) [4], radioimmunoassay [5], chemiluminescence immunoassay [6], and chemiluminescent enzyme immunoassay [7] are the conventional methods for CEA quantification. However, these methodologies are mainly based on expensive devices and time-consuming procedures, and require skilled personnel, hindering their widespread application for patient monitoring—especially at the point-of-care, as well as in resource-limited settings. Therefore, the development of easy-to-use, fast, affordable, but efficient cancer diagnostic methods obviating the need for sophisticated, expensive, bulky equipment and skilled technicians is still in high demand [8,9].

The ASSURED criteria, proposed by the World Health Organization (WHO) in 2003 [10], are a collection of conditions for the ideal tests that can be employed at all levels of health care systems. These criteria emphasize that the ideal diagnostic devices should be affordable, sensitive, specific, user-friendly, rapid and robust, equipment-free, and deliverable to the end-users. However, subsequently, Peeling et al. added two further criteria—real-time connectivity (R), and ease of specimen collection (E)—to the aforementioned conditions, so that these REASSURED criteria enable real-time monitoring of patients by diagnostic systems and enhance the efficiency of health care systems [11]. In this context, lateral flow immunoassays (LFIAs) are simple-to-use immunochromatographic test strip devices with broad applications in clinical analysis, food safety control, environmental monitoring, and drug-abuse assessment [12], which satisfy the WHO’s ASSURED criteria to some extent. Interestingly, the integration of smartphone technology with LFIAs can effectively promote their further potential applications through meeting the real-time connectivity (R) criterion. Benefiting from simplicity and portability, a plethora of smartphone-based (bio)sensing platforms have been reported for the detection of various analytes [13]; however, little attention has been paid to the detection of cancer biomarkers [8]. Although some LFIAs have been developed recently for CEA quantification using quantum dot beads [14,15] and magnetic nanoparticles [16,17] as tags, those studies do not satisfy the REASSURED criteria completely. Meanwhile, despite the broad applications of bare gold nanoparticles (GNPs) as the most straightforward tags in LFIAs, these tags suffer from two traits: (1) the low efficiency of formed immunocomplexes due to the random orientation of antibodies (Abs) prepared by passive adsorption, and (2) their bright-red color, which impedes their visual detection and the interpretation of results—especially in target concentrations near to the limit of quantification—even for analysis via strip readers [18]. To remedy this issue, the traditional strategy involves the application of thiol-containing linkers that bind spontaneously to the surface of GNPs and remain an active carboxyl or amine group for subsequent covalent coupling to Abs [19]. The other approach is the utilization of functional polymers for the encapsulation of GNPs, which not only stabilize nanoparticles, but also provide an active layer for subsequent immobilization of Abs. In this regard, Xu et al. [20] modified the surface of GNPs with a polydopamine layer (GNP@PDA) and used it in a competitive-type LFIA for the sensitive detection of zearalenone in maize, where there was an indirect relation between the visual signal and the target concentration. To the best of our knowledge, the efficiency and behavior of the GNP@PDA tag has not been investigated in a sandwich-type LFIA, and its advantageous quantification via smartphone-based module has not yet been reported.

Herein, a highly sensitive sandwich-type GNP@PDA-based LFIA was developed for the quantification of CEA in sera. The GNPs were first synthesized via the Turkevich method, and then coated with a nanolayer of polydopamine, via the self-polymerization of dopamine in alkaline media, to act as an antibody immobilization layer. By employing GNP@PDA as a tag, in the presence of CEA, sandwich immunocomplexes were formed at the test zone, providing a slightly dark-red color proportional to the concentration of the target. In addition, the fabricated plasmonic LFIA was further coupled with a 3D-printed smartphone-based colorimetric imaging device to capture digital images of the strips’ test zones and, subsequently, to quantify the target concentration via a detection algorithm with a self-developed smartphone app. The developed platform provides a cost-effective, easy-to-use, portable smartphone-based LFIA kit for the quantification of CEA in serum samples down to 0.05 nM, and satisfies the REASSURED criteria, for which it needs only to capture an image of the test zone to show the respective concentration of the target in a sample.

## 2. Materials and Methods

### 2.1. Reagents and Instruments

Hydrogen tetrachloroaurate (III) hydrate (HAuCl_4_·3H_2_O), trisodium citrate (Na_3_C_6_H_5_O_7_·2H_2_O), Tween-20, bovine serum albumin (BSA), and dopamine hydrochloride (DA·HCl) were all purchased from Sigma-Aldrich. The MF1 membrane was obtained from Whatman International Ltd. (Maidstone, UK). The mouse monoclonal antibody (MAB1393) and goat polyclonal antibody (PAB7939)—both against human CEA—were obtained from Abnova (Taipei City, Taiwan). Based on the manufacturer’s information, these Abs are specific to human CEA, and have no cross-reactivity with human nonspecific cross-reacting antigen (NCA, NCA2) or biliary glycoprotein-l (BGP1). The stock solutions of CEA involved in the CEA ELISA kit (MONOKIT)—solely or by dilution—were used as targets. For the preparation of all solutions, Milli-Q-grade water was used. The components of the strips—including pads (SP08, SCL0020215), nitrocellulose membrane (LFNC-C-SS03-15 μm, JCN476015), and backing card (type-L)—were all acquired from Nupore Filtration Systems Pvt. Ltd. (Ghaziabad, India).

The UV–Vis spectra of the solutions were recorded using Cytation 5 (BioTek, Winooski, VT, USA) with a quartz microplate. The structure of synthesized GNP@PDA was observed via transmission electron microscopy (TEM) with a 100 kV running voltage (Zeiss-EM10C-Germany, Jena, Germany). The ζ-potentials were acquired using a Zetasizer Nano ZS (Malvern Instruments, Malvern, UK). A smartphone containing a 13 MP rear camera (Samsung galaxy C8) was utilized for image capturing and analysis. The Fourier-transform infrared–attenuated total reflectance (FTIR–ATR) spectra were measured using an AVATAR (Thermo Scientific, Minneapolis, MN, USA).

### 2.2. Synthesis of GNP@PDA

The GNPs were firstly synthesized via the conventional Turkevich method [21]. In brief, 50 mL of 0.01% HAuCl_4_ solution was heated until boiling, and then 5 mL of 0.04 mol/L sodium citrate was quickly added to it under stirring. After heating for 10 min, the mixture was cooled down to room temperature with constant stirring. The obtained colloidal gold was filtered with a 0.22 μm syringe filter and then kept in a dark bottle at 4 °C.

The GNP@PDA was then synthesized as stated by an earlier work [20], with some modifications. In brief, the pH of the prepared GNPs (1 mL) was set to 7.5 by adding 0.1 mol/L K_2_CO_3_ solution, followed by the addition of 5 μL of 3% H_2_O_2_. Following vigorous stirring for 5 min, 5 μL of freshly prepared DA·HCl solution (10 mg/mL) was added to the solution and stirred for 1 h. The addition of dopamine and stirring were repeated in order to control the graft polymerization of dopamine on the GNPs surfaces. After centrifugation at 9000 rpm for 20 min and removal of the supernatant, the prepared GNP@PDA was washed twice and re-suspended in borate buffer (0.005 mol/L, pH 7.5).

### 2.3. Preparation of GNP–mAb and GNP@PDA–mAb Conjugates

The mouse monoclonal antibody against human CEA (mAb) was conjugated to the GNPs (OD = 1) or corresponding GNP@PDA via simple mixing [20]. To this end, different values of mAb were added dropwise to 1 mL of GNPs or GNP@PDA solution, and after gentle shaking at 4 °C overnight, the mixture was blocked with 2% BSA for 1 h. Subsequently, via centrifugation at 9000 rpm for 20 min, the conjugates were re-suspended in 50 μL of 0.005 mol/L borate buffer containing 5% sucrose and 1% BSA. The conjugates were then kept in microtubes at 4 °C for further use.

### 2.4. Preparation of the Lateral Flow Test Strips

Before assembling the strip components, the sample pad and conjugate pad were pre-treated by dipping them in blocking solutions (0.005 mol/L borate buffer pH 7.5, 0.05% Tween-20 for the former, and the same solution with 3% sucrose for the latter). The pads were then dried out in an oven at 50 °C for 2 h. Thereafter, considering an overlap of 2 mm between the pads and the NC membrane, they were subsequently laminated onto the backing card. Finally, the assembly was cut into strips with a 3.5 mm width. The lengths of the sample pad, conjugate pad, NC membrane, and adsorption pad were 30, 5, 20, and 27 mm, respectively. The desired value of the conjugate was pipetted onto the conjugate pad in each test. For the test zone (TZ), 0.2 μL of polyclonal antibody (pAb) (0.25, 0.5, 1 mg/mL), and for the control zone (CZ), 0.2 μL of 100 μg/mL Protein G (PG), were pipetted. The ready-to-test strips were then dried at room temperature for 1 h and stored for up to one week.

### 2.5. The Fabrication and Setup of the Smartphone-Based Colorimetric Imaging Device

The smartphone-based colorimetric imaging device was composed of two major constituents: a strip cartridge, and an optical imaging box containing a cartridge-placing section, USB cable, and smartphone-reading section (Figure S1). The strip cartridge was designed to hold the strips within the imaging box, with reproducible sections of the TZ being imaged with the camera. The optical imaging box was also composed of an internal light source (high-power white LED, TOP-1BD1, from Epileds, Taiwan, with λ = 7000 nm, light intensity 100–140 Lm, and forward voltage 3–3.4 V) and an electric circuit fixed on a stand powered by municipal electricity along with an adaptor, or by the smartphone itself; it was made with a 3D printer using acrylonitrile butadiene styrene polymer. The 3D CAD file of the platform was first prepared using SolidWorks software. More details of the design and dimensions are given in the Supplementary Materials (Figure S2). The total cost of this platform was USD ~2.5 (Table S1).

### 2.6. The App Development

The smartphone app—TBZMed Sensor (Figure S3)—was planned for the Android platform (version 7.1.1) in the Android studio environment and installed on a Samsung Galaxy C8 smartphone. The app benefits from a user-friendly interface (Figure S3a,b) for selecting and cropping the images of the test zone. For imaging, the strip was placed in the strip cartridge and then inserted in the cartridge-placing section within the imaging box. The light source was then turned on and an image was captured after setting the camera to manual mode, autofocus, and zoom to 4×, and clicking on the strip on the screen. After capturing the image and cropping the test zone with the same area for all samples, the TBZMed Sensor extracted the RGB and grayscale values from the JPEG digital image. Therefore, a calibration curve was first established based on the relationship between the concentration of CEA in standard serum samples and the grayscale value of the TZ. To this end, the area of interest within a TZ could be selected by a circle frame (Figure S3c), and the color information of the selected area was then recorded. The mean values of the R, G, B, and grayscale channels were calculated automatically. The calibration graphs for standard serum samples were investigated and calculated in Microsoft Excel based on different R, G, B, and grayscale data. Then, the best calibration equation was obtained, and was entered into the TBZMed Sensor environment. The channel type was also saved in the app environment (Figure S3d). Finally, the concentration of CEA in an unknown sample could be calculated easily in ng/mL using the saved calibration curve in the app, by simply capturing an image from the TZ and selecting the equation from the app (Figure S3e). In addition, an image of the test result could be easily shared by clinking on the sharing icon (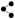) for possible real-time connectivity (Figure S3f).

### 2.7. Lateral Flow Immunoassay Procedure

The standard CEA solutions, or dilutions thereof, were used as targets by mixing them with buffer or standard serum samples. Firstly, the assay procedure consisted of dispensing 5 μL of sample solution onto the sample pad and then adding 60 μL of running buffer (Tris buffer 0.05 mol/L, 0.05% Tween-20). After complete running of the test, images were captured by the smartphone-based platform and then analyzed via the developed app. The data were represented as mean ± SD for three replicates. The calibration graph obtained for the serum samples was used for insertion into the app environment and reading the concentrations of clinical samples. For serum and clinical samples, the sample pad’s performance was improved by putting the MF1 membrane on it. According to the manufacturer’s information, this pad is a bound glass-fiber filter that can be used for lateral flow assays, and is typically used for whole-blood analysis.

### 2.8. Clinical Samples Analysis

This study was approved by the Medical Ethics Committee of Tabriz University of Medical Sciences, Iran, and all methods were performed following the relevant guidelines and regulations. The clinical serum samples were obtained from Shahid Ghazi Tabatabaei Hospital of Tabriz, Iran, and included 12 positive samples and 14 negative samples. Real and false negative samples were determined according to a report from the hospital during a one-year follow-up of the patients’ conditions. The assay procedure for clinical sample analysis was as described in Section 2.7. The values of CEA in clinical samples were measured using a CEA ELISA kit (MONOKIT) in the hospital laboratory.

## 3. Results and Discussion

**Scheme 1:**
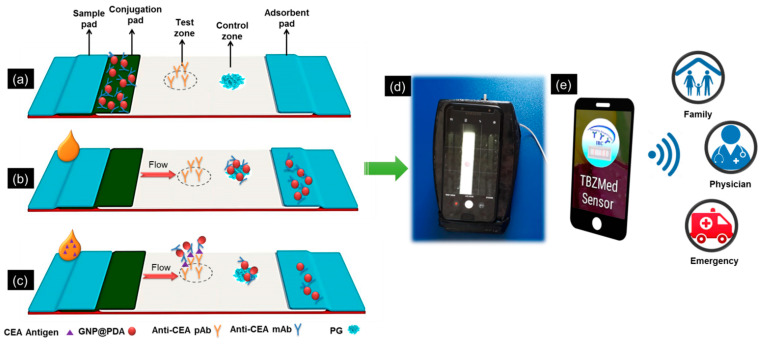
Schematics of (**a**) ready-to-use test strip and its components, with GNP@PDA–mAb conjugates deposited on the conjugate pad, pAb on the test zone, and PG on the control zone; (**b**) the formation of GNP@PDA–mAb–PG immuno-sandwich structures in the control zone in the absence of CEA; (**c**) the formation of a GNP@PDA–mAb–CEA–pAb immunocomplex at the test zone and GNP@PDA–mAb–PG at control zone in the presence of CEA; (**d**) smartphone-based quantification and (**e**) real-time connectivity using our developed app.

Scheme 1 displays the schematic diagram of CEA detection by the GNP@PDA-based lateral flow immunosensor and its components (Scheme 1a). In the absence of CEA (Scheme 1b), only a slightly dark red at the CZ was formed for the conjugate bound to PG; however, in the presence of CEA (Scheme 1c), a GNP@PDA–mAb–CEA–pAb immunocomplex was formed at the TZ, and GNP@PDA–mAb–PG was formed at the CZ; hence, a slightly dark-red color appeared on them. The color intensity at the TZ was recorded with the developed platform. A higher concentration of CEA in the sample resulted in the stronger color intensity of the TZ up to the hook effect region, which then turned to low signal intensities. Eventually, the results can be easily quantified via the smartphone readout and subsequently shared via the Internet with a physician, family, and or even emergency services (Scheme 1d).

### 3.1. Characterization of GNP–mAb and GNP@PDA–mAb Conjugates

The physicochemical properties of nanoparticles and nanobioconjugates have a considerable effect on the performance of the test strip. The full characterization of these nanostructures is given in the Supplementary Materials, Section S2 and Figure S4, showing successful functionalization of GNPs by a nanometer layer of PDA. The average ζ-potential for GNPs is −15.6 ± 1.4 mV, which is reduced by the formation of the PDA layer around them, reaching as low as −29.2 ± 2.3 mV. This is mainly attributed to the abundance of hydroxyl groups in the PDA layer [20]. After the immobilization of Abs on the nanoparticles’ surface, due to the positive charge of Abs at pH 7.5, the ζ-potential is increased slightly (−28.0 ± 1.6 mV). Using Tukey’s multiple comparisons test clearly shows significant differences between the zeta potential values obtained for all materials except for GNP@PDA and GNP@PDA–Ab, which showed no significant statistical differences (*p*-value = 0.7095) (See Section S2, Table S2 in the Supplementary Materials), possibly due to the small values of Ab compared to the PDA layer. The TEM image of GNP@PDA (Figure S4f) shows a thin layer of PDA with lower contrast (~2 nm) around the GNPs, indicating that the structure of the GNP@PDA is core–shell with an average diameter of ~27.49 ± 7.6 nm.

### 3.2. Optimization of Effective Factors on the Performance of the Developed Smartphone-Based LFIA Kit

The blocking of sample and conjugation pads, the type of running buffer, the amount of mAb immobilized on the GNP@PDA, the amount of GNP@PDA–mAb dropped on the conjugation pad, the amount of pAb at the TZ, and the reading time are some of the most important factors that impact on the performance of our developed LFIA kit. Hence, the effects of the mentioned parameters were investigated and, subsequently, the optimal levels were used for the next experiments. The optimization results and further descriptions are given in Section S3 of the Supplementary Materials, and in Figures S5 and S6. The results show that blocking of the sample pad with 0.005 mol/L borate buffer pH 7.5 containing 0.05% Tween-20, and the conjugate pad with 0.005 mol/L borate buffer pH 7.5 containing 0.05% Tween-20 and 3% sucrose, are necessary for the successful functioning of the strips. Among the three buffers—including borate (0.005 mol/L, pH 7.5), phosphate (1X, pH 7.4), and Tris (0.005 mol/L, pH 7.5), all with 0.1% Tween-20—the third one showed a good flow rate and the least nonspecific adsorption of conjugates on the TZ. The amount of mAb immobilized on GNP@PDA affects the immunosensor performance in two ways: Firstly, sufficient mAb on the nanoparticles is necessary in order to be able to form immuno-sandwich complexes on the TZ. Secondly, the high amounts of mAb cause steric hindrance, or even capture more target molecules and prevent their immunocomplex formation within the TZ [22]. The values of 3.2 and 6.41 μg per mL of GNPs/PDA showed favorable behavior in the analysis of buffer and serum samples, respectively. The volume of the conjugate also played a critical role in the assay—by its increase, the signal at the TZ was increased, but the background signal was also boosted. Hence, 2.5 μL of the probe was selected as the optimal level. The amount of pAb at the TZ is essential for the efficient capturing of GNP@PDA–mAb–CEA immunocomplexes; its performance was increased until the advent of steric hindrance at 0.5 mg/mL, which reduced the immuno-sandwich formation. Therefore, 0.5 mg/mL was selected as the optimal value for the amount of pAb at the TZ. Since the contrast of the TZ was decreased over time because of the drying of the membrane, the images should be captured within 2 min after completion of the test. For better comparison, the performance of GNPs as tags was examined under the same conditions obtained for GNP@PDA.

### 3.3. Analytical Performance of the Developed Smartphone-Based LFIA Kit

**Figure 1:**
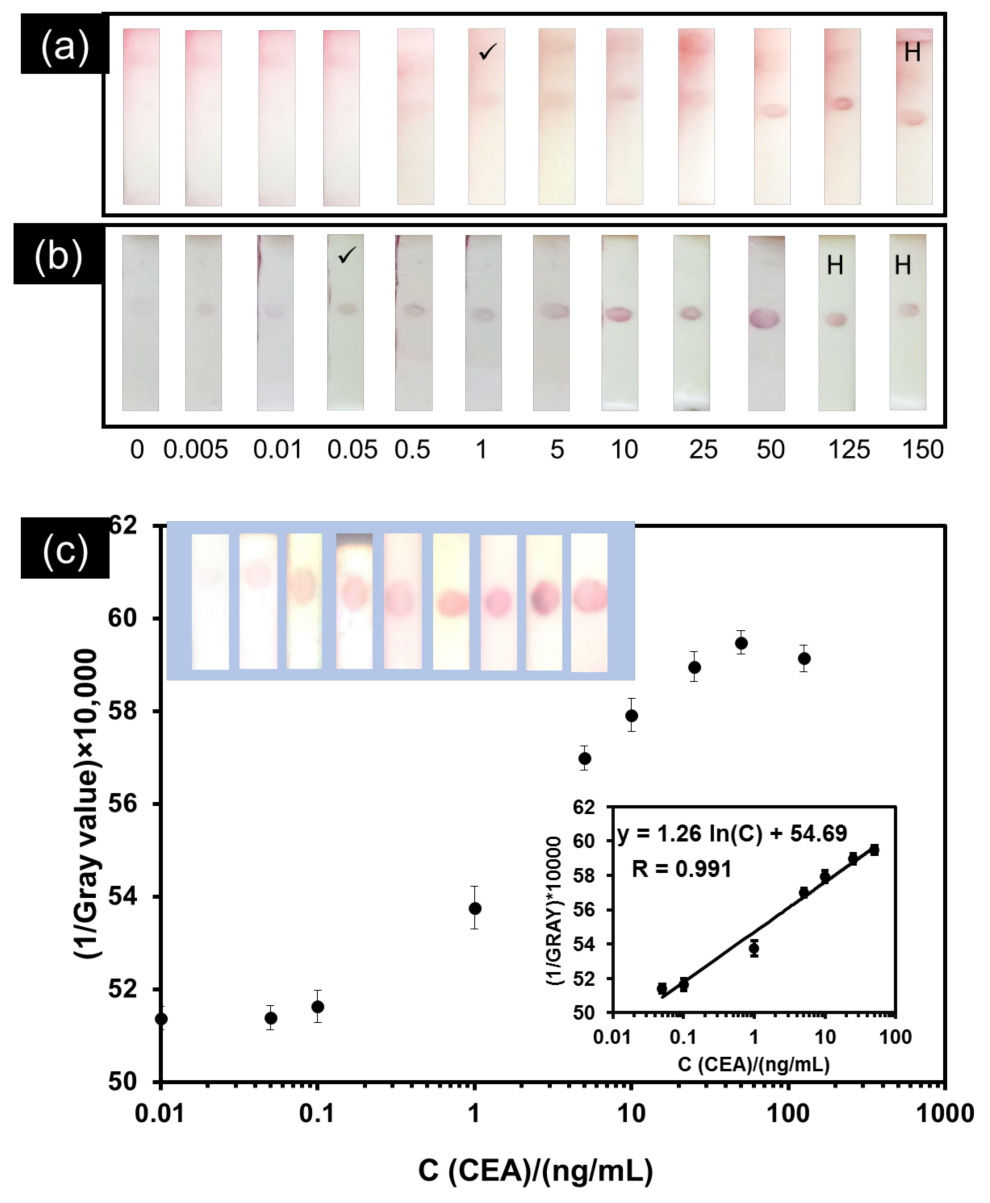
Images of strips taken without the imaging platform: (**a**) GNPs and (**b**) GNP@PDA as tags for different values of CEA in buffer solution (in ng/mL), (√ shows the visual limit of detection, H represents the occurrence of the hook effect), and (**c**) (1/Gray value)* × 10,000 vs. concentration of CEA (ng/mL) in standard serum samples. Insets: calibration curve for analysis of CEA, and images of strips taken with the imaging platform.

The citrate-capped GNPs show bright-red color; in contrast, the color of GNP@PDA is slightly darker due to the black color of PDA self-polymerized by dopamine on GNPs in an alkaline medium. Moreover, the PDA layer enables more efficient immobilization of Ab on the tags’ surface. These advantageous features can boost the signal intensity and detection sensitivity. To check this, a comparison was first made between the utility of GNPs and GNP@PDA tags in the visual analysis of CEA via the strips in the buffer. Under the optimized conditions, the same volumes of CEA at various concentrations were added to the strips, and the visual signals from the TZ were assessed. As seen in Figure 1a,b, the dots formed at the TZ for strips prepared using GNP@PDA as a tag have a slightly darker red color compared to strips prepared using GNPs alone and, interestingly, the visual limits of detection for GNPs and GNP@PDA as tags are 1 and 0.05 ng/mL, respectively. This observation proves the better efficiency of GNP@PDA as tags in a sandwich-type lateral flow test strip compared to GNPs, with 20 times reduction in the visual limit of detection. Another advantage of GNP@PDA compared to GNPs is its better surface blocking and, hence, lower background signal, which enhances the sensitivity and reduces the limit of detection. Such improved blocking behavior may be a result of the interaction of BSA with PDA chains anchored on the surface of GNPs. The hook effect was observed for concentrations higher than 125 ng/mL for GNPs, compared to 50 ng/mL for GNP@PDA. This is a common phenomenon in sandwich-type LFIAs because of the occupation of fragment antigen-binding sites for both capture and reporting of Abs in the presence of high concentrations of the target, which reduces the immunocomplex formation and, hence, deceases the color of the TZ [23]. This is also an important issue in the use of commercial CEA strips. As for commercial strips, herein, in the case of suspicious samples, the user could dilute the sample and re-check it by strip. If the signal increased after dilution of the sample, this showed the high concentration of CEA and the occurrence of the hook effect. Therefore, the user should consider the result obtained for the diluted sample to be a correct answer.

Visual detection of LFIA results suffers from some limitations: firstly, the user’s eyesight impacts on the reading of the TZ, and can become challenging, especially at low concentrations of analyte; secondly, different environmental illumination conditions affect the interpretation of colors and results [24,25]. However, the imaging box developed herein provides constant lightning conditions, and the smartphone quantification platform provides quantitative results in a facile way, preventing the user’s eyesight error. Since the matrix of real samples is more complex than the buffer, the same quantitative studies performed with the buffer were conducted in standard serum samples containing the desired values of CEA, with GNP@PDA as a tag, to obtain the desired calibration curve. The images of strips and the calibration curve are given in Figure 1c. After elucidating various calibration curves for the color intensities of the R, G, B, and Gray channels vs. the concentration of the target, as seen in Figure 1c, a logarithmic relationship was obtained between the concentration of CEA in serum and the (1/Gray value)*10,000 in the range of 0.05–50 ng/mL with R = 0.99. The relative standard deviation values were in the range of 2.5% to 12.5%, confirming the good reproducibility of the results. For concentrations higher than 50 ng/mL, the hook effect was observed. Trials for concentrations lower than 0.05 ng/mL failed to show considerable changes compared to the buffer, clearly showing the effect of corona formation in serum samples, as stated elsewhere [26].

Table 1 indicates the intra-assay and inter-assay recoveries for samples spiked with CEA at different concentrations using GNP@PDA as a tag. The experiments were carried out in triplicate for each spiked concentration. The intra-assay studies were performed within 1 day, and the inter-assay studies were conducted for 3 days. The average recoveries and coefficient of variation (CV) values for the intra-assay tests were within 95–120% and 6.3–10%, respectively. The inter-assay recoveries ranged from 90 to 120%, with a CV ranging from 8.33 to 11.66%. These results prove that this immunosensor benefits from high accuracy and precision [27]. Since long-term stability studies were not the purpose of our work, we did not check it, but taking into account the several weeks of stability of commercially available pregnancy tests, we can expect similar performance [28].

### 3.4. Application of the Developed Smartphone-Based LFIA Kit for the Detection of CEA in Clinical Serum Samples

**Figure 2:**
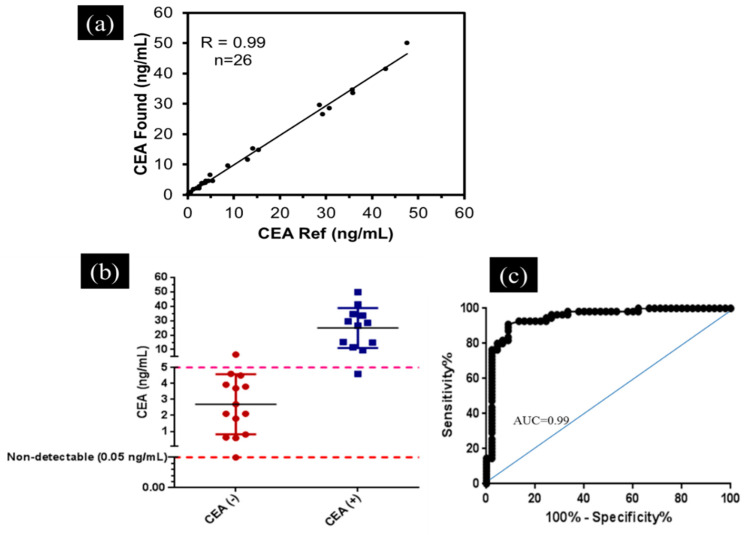
(**a**) Comparison of the results obtained with the developed smartphone-based LFIA kit (CEA found) and CEA reference (commercial ELISA kit) in the measurement of CEA in clinical samples. (**b**) The estimated CEA levels corresponding to each sample, resulting from the analysis facilitated by the smartphone-based platform. The clinical serum samples were classified in two groups: CEA-positive (+, n = 12 samples), and CEA-negative (−, n = 14 samples). (**c**) ROC analysis, with area under the curve (AUC) of 0.99.

After quantifying CEA in real serum samples, the extracted calibration curve was inserted into the developed “TBZMed Sensor” app and used to analyze the clinical serum samples. Among these samples, 12 samples had CEA values higher than 5 ng/mL (positive samples), while 14 samples had CEA values lower than 5 ng/mL (negative samples). A comparison of the results obtained by our developed smartphone-based LFIA kit and ELISA is given in Figure 2a. As seen, an excellent correlation (R = 0.99) between these two approaches was attained, confirming a good agreement between them. In addition, the sensitivity and specificity of the developed smartphone-based LFIA kit were further calculated. The obtained results (sensitivity of 91% and specificity of 93%) in Table 2 and Figure 2a illustrate that the developed smartphone-based LFIA kit can potentially be utilized for accurate and rapid monitoring of cancer treatment at sites far from centralized laboratory facilities. Using an unpaired *t*-test for the analysis of CEA (+) and CEA (−) clinical samples revealed a considerable ability of the developed platform to discriminate between these two groups (*p*-value < 0.0001) (Figure 2b). Receiver operating characteristic (ROC) analysis is a valuable means of assessing the performance of diagnostic tests, acting as a simple graphical tool for displaying the accuracy of a medical diagnostic test. ROC analysis was also carried out on clinical serum samples. The TP, TN, FP, and FN values were obtained according to a report from the hospital during a one-year follow-up of the patients’ conditions [29]. The area under the ROC curve is a summary measure that essentially averages diagnostic accuracy across the spectrum of test values. As seen in Figure 2c, with an area of 0.99, the developed platform benefits from high diagnostic accuracy [30].

A comparison was also made between our developed cost-effective smartphone-based LFIA kit and currently reported lateral-flow-based portable detection systems for the quantification of CEA, in terms of analytical performance and meeting the REASSURED criteria (Table 3). Since the commercial magnetic and fluorescent strip readers do not have ability to connect to the Internet, such assays suffer in terms of real-time connectivity (lack of the R criterion), as described for [14,17] in Table 3. The approaches developed in [16,17] may suffer in terms of the user-friendly (U), equipment-free (E), and deliverable to the end-user (D) criteria due to the complexity of the quantification procedure. In addition, because of the use of external image analysis software, [16] could not satisfy the necessity of easy quantification and sharing via smartphone (lack of the R criterion). The complexity of the quantification process is also another drawback of some previous works, such as [15,16,31] (lack of the E and U criteria). As seen in Table 2 and Table 3, with a comparable linear range of 0.05–50 ng/mL, considerable sensitivity and specificity of 91% and 93%, respectively, the capability of analyzing serum samples and potential application to blood samples, easy access to the Internet for transferring results via the developed app, low cost of the assembly (USD ~2.5, see Table S1), and its user friendly characteristics—i.e., quantification of CEA in clinical samples by image capturing alone—this device satisfies the REASSURED criteria [32,33,34]. Although our developed platform benefits from the REASSURED criteria, it may suffer from camera-dependent output results, which is an intrinsic drawback of smartphone-based quantification systems. To remedy this issue, a simple approach is to attach the smartphone or a camera to the black box system, providing all users with the same image capturing system.

## 4. Conclusions

In conclusion, by combining the desired characteristics of GNP@PDA as an efficient tag in a sandwich-type LFIA, and by the development of a portable smartphone-based platform, a simple, cost-effective (USD ~2.5, see Table S1), and easy-to-use immunosensing device was developed for the smartphone-based detection and quantification of CEA in human serum. With a linear range of 0.05–50 ng/mL and a low LOD of 0.05 ng/mL, sensitivity of 91%, and specificity of 93%, the developed smartphone-based LFIA kit provides the desired characteristics for point-of-care evaluation of cancer biomarkers. The linear range of the strip satisfies the requirements for clinical analysis. The employed MF1 membrane in the sample pad provides the capability to perform whole-blood-sample analysis; hence, our developed sensing platform can potentially be employed for blood sample tests without any pretreatment or enrichment, addressing the REASSURED criteria. In addition to the desired characteristics of the platform, the automation of image capturing via our developed app may further improve utility and user-friendliness. Building upon the satisfactory results of our developed smartphone-based LFIA platform in the detection of CEA in human serum samples—especially in comparison with the results of reference methods—and its other advantageous features as an assay kit that meets the World Health Organization’s REASSURED criteria, we believe it could potentially be widely exploited for patient monitoring, particularly at sites far from centralized laboratory facilities, for point-of-care applications, and in resource-limited settings. Although our developed platform shows some benefits in terms of the REASSURED criteria, it should be noted that this system may suffer from camera-dependent output results, which is an intrinsic drawback of smartphone-based quantification systems. To remedy this issue, there are two solutions: (1) some calibration samples can be required within the final kit to calibrate the quantification module before analyzing a real sample, due to differences in the optics of the camera and the qualities of the employed CCDs; (2) a camera can be attached to the black box system, which provides all users with the same image capturing system. Our group is also working in this area to offer solutions to this drawback.

## Figures and Tables

**Table 1:** The obtained recovery and coefficient of variation (CV) results for CEA-spiked serum samples using the developed kit.

Spiked CEA (ng/mL)	Intra-Assay			Inter-Assay		
	Mean ± SD	Recovery (%)	CV (%)	Mean ± SD	Recovery (%)	CV (%)
0.5	0.6 ± 0.06	120	10	0.6 ± 0.07	120	11.66
2	2.3 ± 0.15	115	6.5	2.4 ± 0.25	120	10.41
20	19 ± 1.2	95	6.3	18 ± 1.5	90	8.33
35	34 ± 2.6	97	7.6	35 ± 3.4	100	9.70
45	46 ± 3.4	102	7.4	46 ± 5.2	102	11.30

**Table 2:** Characteristics of the developed smartphone-based LFIA kit in the analysis of clinical serum samples.

Samples	Number of Positive Results	Number of Negative Results	Characteristic Parameter	
12 (+)	11	1	Sensitivity	91%
14 (−)	1	13	Specificity	93%

Sensitivity = (TP/TP + FN)∗100; specificity = (TN/TN + FP)∗100; TP: true positive; FN: false negative; TN: true negative; FP: false positive.

**Table 3:** Comparison of some characteristics of our developed smartphone-based LFIA kit with recently reported LFIA-based portable systems, in terms of the quantification of CEA.

Detection Strategy	Used Tag	LR * (ng/mL)	LOD ** (ng/mL)	Detection Time (min)	REASSURED * Criteria	Ref.
Commercial magnetic strip reader	Magnetic particles	1–100	0.045	30	-EASS-R- -	[17]
Smartphone-based colorimetric image analysis	Magnetic NPs containing Ab and biotinylated DNA	0.25–100	0.0375	15	-EASS-R- -	[16]
Fluorescent handing system	Quantum dots	1–100	5	20	REASS-R-D	[15]
Fluorescent handing system	Quantum dot nanobeads	1–50	0.049	15	REASS-R-D	[32]
Commercial fluorescent reader	Quantum-dot-doped polystyrene nanoparticles	2.8–680	0.35	15	-EASSURED	[14]
Smartphone-based colorimetric image analysis	GNP@PDA	0.05–50	0.05	15	REASSURED	this work

* LR: linear range; ** LOD: limit of detection; * REASSURED criteria: real-time connectivity (R), ease of specimen collection (E), affordable (A), sensitive (S), specific (S), user-friendly (U), rapid and robust (R), equipment-free (E), deliverable to the end-user (D).

## Data Availability

Not applicable.

## Supplementary Materials

The following are available online at https://www.mdpi.com/article/10.3390/bios11100392/s1: Section S1: Figure S1: Photos of smartphone-based colorimetric imaging device: (a) strip cartridge containing a test strip, the location of the cartridge-placing section within the imaging box, and the USB cable; (b) the location of the smartphone holder and imaging aperture; (c) the whole assembly of the platform, showing the placed strip within the box and the electric circuit connected to the smartphone, and imaging of the strip; Figure S2: (a) photo; (b) design and dimensions of 3D-printed LED holder—i: hole for placing LED; ii: cartridge-placing section. Illustration and dimensions of (c) strip cartridge, and (d,e) imaging box; Table S1: Estimated cost of fabrication of each imaging platform; Figure S3: The main interface of TBZMed Sensor: (a) icon; (b) the main interface for selecting an image from the gallery or taking an image via the camera; (c) selecting the test zone and cropping the image; (d) selecting the calibration equation; (e) reading the concentration of CEA in ng/mL; and (f) real-time sharing of the test result; Section S2: Characterization of GNP-mAb and GNP@PDA-mAb conjugates; Figure S4: Characterization of nanostructures, UV–Vis spectra of different nanoparticles and respective bioconjugates: (a) initial; (b) in the presence of 1% NaCl; (c) numerical values of λmax; (d) FTIR–ATR spectra of GNPs and GNP@PDA; (e) ζ-potential values; (f) TEM image of GNP@PDA; Table S2: Results of Tukey’s multiple comparisons test on ζ-potential values; Section S3: Optimization of effective factors on the performance of the developed smartphone-based LFIA kit; Figure S5: The flow behavior of strips containing (a) unblocked conjugate pad, (b) blocked conjugate pad (in lateral flow format), (c) both the sample and conjugate pads blocked, and (d) unblocked sample and conjugate pads (in dipstick format); Figure S6: (a) Effect of running buffer on strip performance—T depicts Tris (0.005 mol/L, pH 7.5), B depicts borate (0.005 mol/L, pH 7.5), and P depicts phosphate (1X, pH 7.4), all with %0.1 Tween-20; 1 and 2 indicate that the concentration of CEA is 0 and 5 ng/mL, respectively. (b) Effect of the amount of mAb immobilized on GNP@PDA, including 1.6, 3.2, and 6.4 μg for buffer sample testing (b1 to b3), and 3.2, 6.4, and 9.6 μg for serum sample testing (s1 to s3) on strip performance. (c) Effect of the volume of GNP@PDA–mAb dropped on the conjugation pad—where V1 to V3 depict 1.5, 2.5, and 5 μL, respectively (in each case the left image is for blank and the right image is for the sample containing CEA 5 ng/mL)—on strip performance. (d) Effect of different amounts of polyclonal antibody immobilized on the test zone on the performance of the strip. C1 to C4 represent concentrations of 0, 0.25, 0.5, and 1 mg/mL, respectively.
