# Supplementary material for: On-Site Detection of Carcinoembryonic Antigen in Human Serum
Source: Biosensors (Basel). 2021 Oct 14;11(10):392. doi: 10.3390/bios11100392 (PMC8534016; doi:10.3390/bios11100392)
Supplement: Supplementary file 1 [file biosensors-11-00392-s001.zip › biosensors-1402490-supplementary.pdf]

Supporting Information

On-site detection of carcinoembryonic antigen in human serum

Tohid Mahmoudi[1], Mohammad Pourhassan-Moghaddam [2,3], Behnaz Shirdel [1], Behzad Baradaran [1], Eden Morales-Narváez [4]* and Hamed Golmohammadi [5]*

[1] Immunology research center, Tabriz University of Medical Sciences, Tabriz, Iran; mahmoodit@tbzmed.ac.ir (T.M), behnaz.shirdel201467@gmail.com (B.Sh)
[2] School of Life Sciences, Faculty of Science, University of Technology Sydney, Sydney, NSW 2007, Australia; m.pourhassan_moghaddam@unsw.edu.au
[3] ARC Research Hub for Integrated Device for End-User Analysis at Low-Levels (IDEAL Research Hub), Faculty of Science, University of Technology Sydney, Sydney, NSW 2007, Australia
[4] Biophotonic Nanosensors Laboratory, Centro de Investigaciones en Óptica, A. C. Loma del Bosque 115, Lomas del Campestre, 37150 León, Guanajuato, Mexico
[5] Nanosensor Bioplatforms Laboratory, Chemistry and Chemical Engineering Research Center of Iran, 14968-13151, Tehran, Iran

* Corresponding authors: E. Morales-Narváez (eden@cio.mx); Hamed Golmohammadi (golmohammadi@ccerci.ac.ir)

Table of Contents

Section S1:

Figure S1 Photos of smartphone-based colorimetric imaging device **(a)** strip cartridge containing a test strip and the location of cartridge placing section within the imaging box and the USB cable, **(b)** the location of smartphone holder and imaging aperture, **(c)** the whole assembly of the platform showing the placed strip within the box and connected the electric circuit to the smartphone, and imaging of the strip ……………….……………….. **Page 3**

Figure S2 (a) photo, **(b)** design and dimensions of 3D printed LED holder i: hole for placing LED, ii: cartridge placing section, Illustration and dimensions of **(c)** strip cartridge, **(d)** and **(e)** imaging box ……………………………………………….…………….………… **Page 4**

Table S1 Estimated cost of fabrication of each imaging platform ……….….…….…. **Page 5**

Figure S3 The main interface of TBZMed sensor, **(a)** icon, **(b)** the main interface for selecting an image from the gallery or taking by the camera, **(c)** selecting the test zone and cropping the image, **(d)** selecting the calibration equation, **(e)** reading the concentration of CEA in ng/mL, and **(f)** real-time sharing of the test result……………….….….…. **Page 6**

Section S2:

Characterization of GNP-mAb and GNP@PDA-mAb conjugates ……………..….. **Page 7**

Figure S4 Characterization of nanostructures, UV-Vis spectra of different nanoparticles and respective bioconjugates **(a)** initial, **(b)** in the presence of 1% NaCl, **(c)** numerical values of λ_{max}, **(d)** FTIR-ATR spectra of GNPs and GNP@PDA, **(e)** ζ-potential values **(f)** TEM image of GNP@PDA……………………...……… **Page 9**

Table S2 Results of Tukey's multiple comparisons test on ζ-potential values ………………………………………………………………………………….. **Page 10**

Section S3:

Optimization of effective factors on the performance of the developed smartphone-based LFIA kit ………………………...………………………………….....………... **Page 11**

Figure S5 The flow behaviors for strips containing **(a)** unblocked conjugate pad, **(b)** blocked conjugate pad (in lateral flow format), and **(c)** both of the sample and conjugate pads have been blocked, **(d)** unblocked sample and conjugate pads (in dipstick format) …..….. **Page 12**

Figure S6 (a) Effect of running buffer on strip performance, T depicts Tris (0.005 mol/L, pH 7.5), B depicts borate (0.005 mol/L, pH 7.5), P depicts phosphate (1X, pH 7.4), all with %0.1 Tween-2. 1 and 2 depict the concentration of CEA is 0 and 5 ng/mL, respectively, **(b)** Effect of the amount of mAb immobilized on GNP@PDA including 1.6, 3.2, 6.4 µg for buffer samples testing (b1 to b3) and 3.2, 6.4, 9.6 µg for serum samples testing (s1 to s3) on strip performance, **(c)** Effect of the volume of GNP@PDA-mAb dropped on conjugation pad including V1 to V3 depict 1.5, 2.5, 5 µL (in each case the left image is for blank and the right image is for the sample containing CEA 5 ng/mL) on strip performance, **(d)** Effect of different amounts of polyclonal antibody immobilized on the test zone on the performance of the strip. The C1 to C4 represent concentration of 0, 0.25, 0.5 and 1 mg/mL, respectively.… ……………………………………………………………………………………**Page 14**

References ....**Page 15**

Section S1:

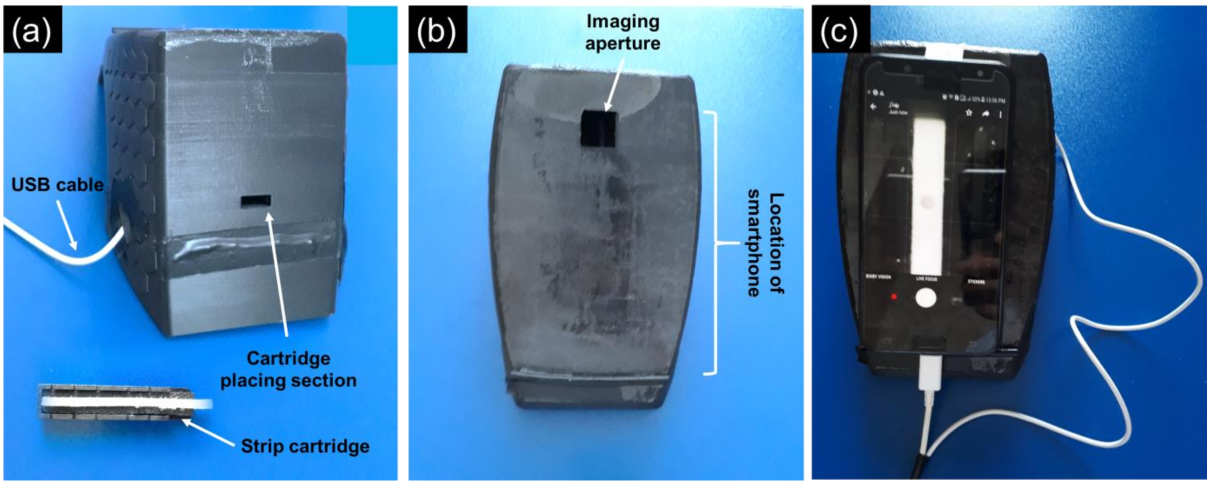

Figure S1 Photos of smartphone-based colorimetric imaging device **(a)** strip cartridge containing a test strip and the location of cartridge placing section within the imaging box and the USB cable, **(b)** the location of smartphone holder and imaging aperture, **(c)** the whole assembly of the platform showing the placed strip within the box and connected the electric circuit to the smartphone, and imaging of the strip

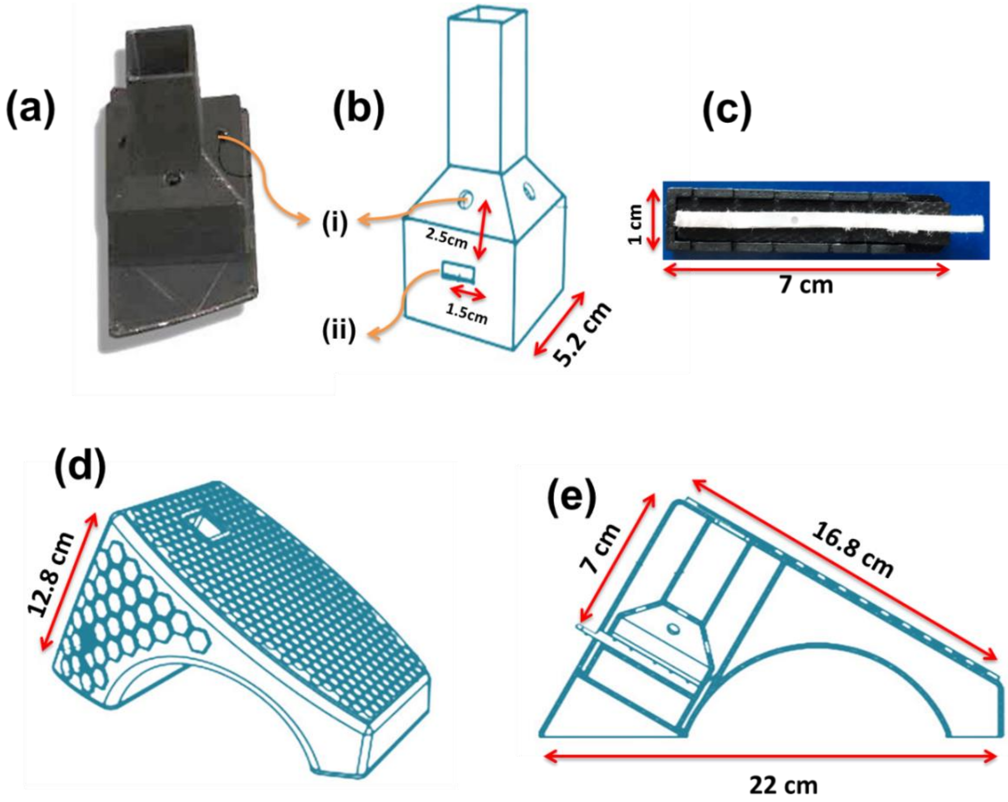

Figure S2 (a) photo, **(b)** design and dimensions of 3D printed LED holder i: hole for placing LED, ii: cartridge placing section, Illustration and dimensions of **(c)** strip cartridge, **(d)** and **(e)** imaging box

Table S1 Estimated cost of fabrication of each imaging platform

**Material**	**Number required**	**Price (USD)**	**Total price (USD)**
LED lamps	2	0.35	0.7
Electrical circuit and cable	1	1	1
Plastic materials (ABS)	0.15 g	2.36/Kg	0.354
Cost of production of plastic parts	1	0.47	0.47
			Total= 2.52

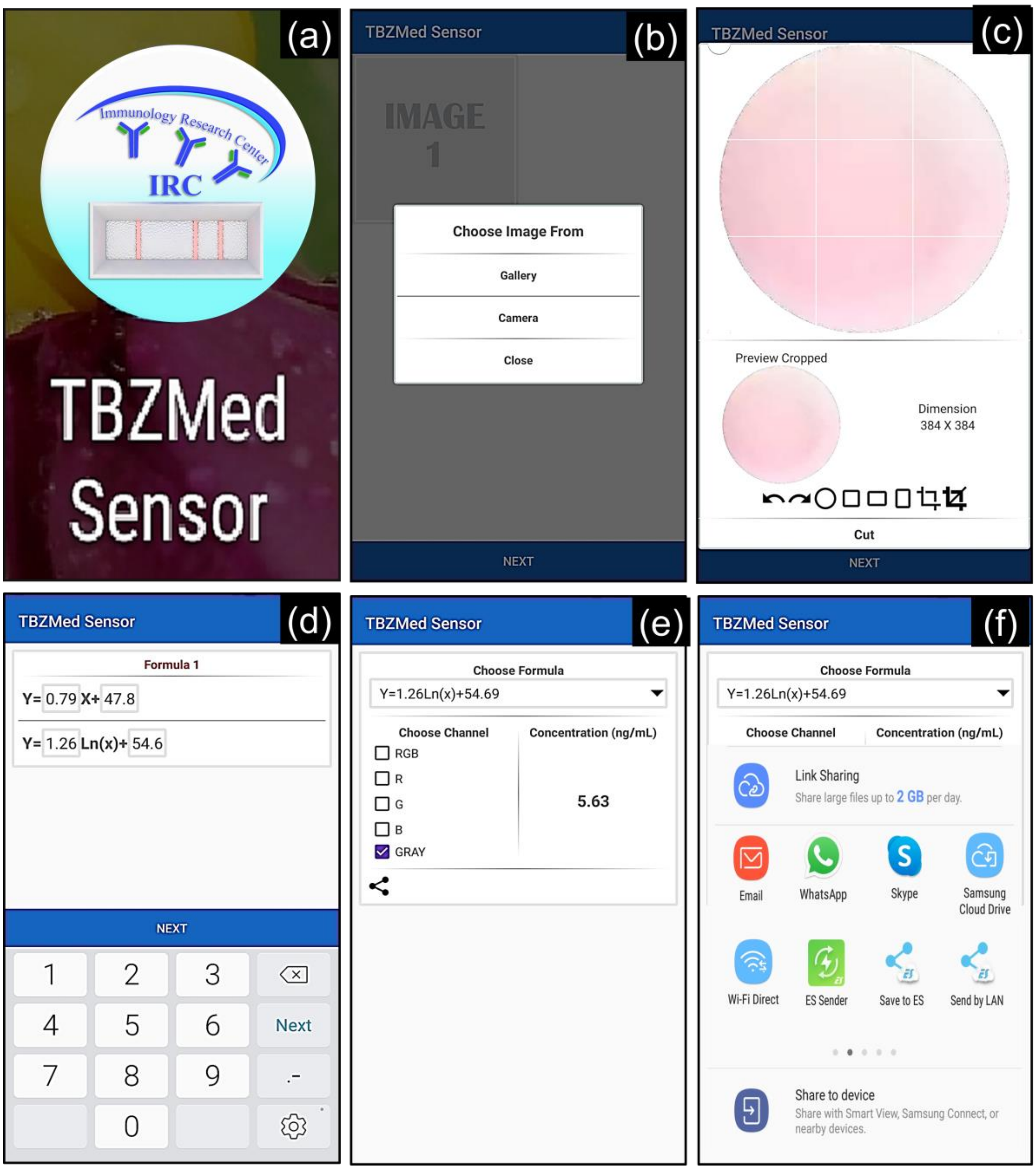

Figure S3 The main interface of TBZMed sensor, **(a)** icon, **(b)** the main interface for selecting an image from the gallery or taking by the camera, **(c)** selecting the test zone and cropping the image, **(d)** selecting the calibration equation, **(e)** reading the concentration of CEA in ng/mL, and **(f)** real-time sharing of the test result

Section S2:

Characterization of GNP-mAb and GNP@PDA-mAb conjugates:

The UV-Vis spectra of bare GNP and GNP@PDA and respective bioconjugates in the absence and presence of NaCl 1% are given in Figure S4 a and b, respectively. The respective surface plasmon resonance (SPR) peaks are also given in Figure S4 c in Table. As seen, the SPR peak for GNPs is located at 525 nm so that by the formation of the PDA layer, the λ_{max} is not changed considerably but the width of the absorption peak is increased, representing the interaction of PDA with nanoparticles surface. Besides, the immobilization of Ab on GNPs surface results in a 2 nm red-shift in λ_{max}, depicting covalent bond formation between reactive amine groups in Ab and ortho-dihydroxy-phenyl functional group within the PDA layer via Michael addition or Schiff base reaction and resulted in increasing the dielectric constant of shell around particles surface [1-3]. In addition, one of the main important factors related to tags employed in LFIAs is their stability in salty environments, in which the more stability of the tag the better its performance. Therefore, the stabilities and aggregation behaviors of citrate-capped GNPs, GNP@PDA, and conjugates were estimated in the presence of 1% NaCl. As seen in Figure S4 b, and c, the GNPs easily aggregate by the addition of salt, and the intensity of the SPR peak at 525 nm is reduced, while a peak at 605 nm is appeared. In contrast, the red shifts for GNP@PDA and GNP@PDA-Ab are 4 and 6 nm, respectively, without the formation of a new peak around 605 nm, showing considerable stability of them. Although the addition of BSA to conjugate did not show any change in the λ_{max}, it results in more stabilization of conjugate, i.e. less red shifts in λ_{max}.

The evidence for the advent of PDA and its respective functional groups on GNPs surface was further attained by FTIR-ATR spectroscopy. As depicted in Figure S4 d, the primary asymmetric and symmetric stretching vibrations of COO^- at around 1598 cm^{-1} and 1416 cm^{-1} in citrate capped GNPs were mostly disappeared, and a new peak attributed to the featured

absorption of stretching vibrations of C=C double bonds in an aromatic ring at 1640 cm^{-1} was appeared, showing successful functionalization and formation of PDA layer on GNPs. In addition, double bands at 3000-3400 cm^{-1} region were ascribed to $-NH_2$ stretching within PDA [4,5]. Besides, the ζ-potential for bare nanoparticles and conjugates are given in Figure S4 e. The average ζ-potential for GNPs is -15.6±1.4 mV, which reduces by the formation of the PDA layer around them and reaches to -29.2±2.3 mV. This is mainly attributed to the plenty of hydroxyl groups in the PDA layer [6]. After immobilization of Abs on nanoparticles surfacethe ζ-potential did not increased from statistical view point (-28.0±1.6 mV). In the case of BSA, it further neutralizes the negative charge of conjugates and the potential value reaches to -22.4±1.9 mV. Hence, the stabilization property of BSA is mainly attributed to the steric hindrances, not electrostatic. Taking a Tukey's multiple comparisons test clearly show significant differences between ζ-potential values obtained for all materials except for GNP@PDA and GNP@PDA-Ab (P value=0.7095), as seen in Table S2. Such insignificant change in ζ-potential values by addition of Ab to GNP@PDA maybe attributed to the little amount of Ab in compared with the formed polymer layer on GNP. Finally, the TEM image of GNP@PDA, Figure S4 f, shows a thin layer of PDA with lower contrast, about 2 nm, around GNPs indicating the structure of the GNP@PDA is core-shell with an average diameter about 27.49 ± 7.6 nm. So, an appropriate thickness of the PDA layer has been attained which enhanced the dispersity of GNPs and provided a functional layer for the subsequent mAb coupling process.

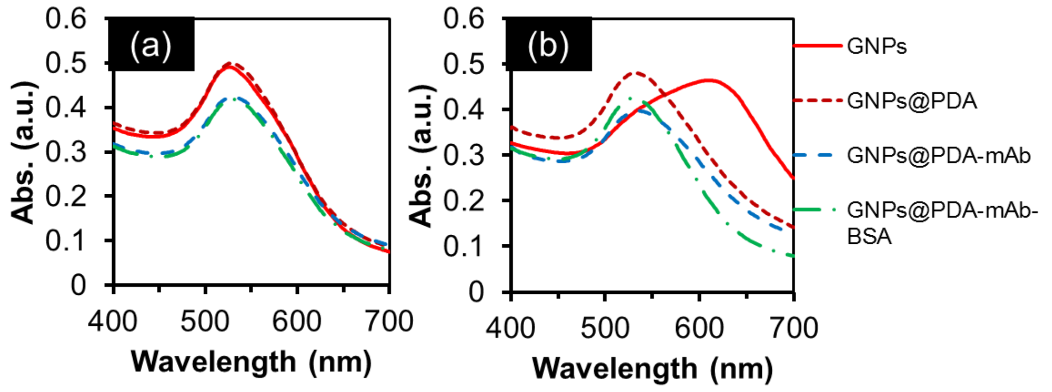

(c)

Nanoparticle	λ_{max} (nm)	
	Initial	In the presence of 1% NaCl
GNPs	525-527	605-614
GNP@PDA	525-531	529-535
GNP@PDA-mAb	527-532	533-536
GNP@PDA-mAb-BSA	527-532	527-533

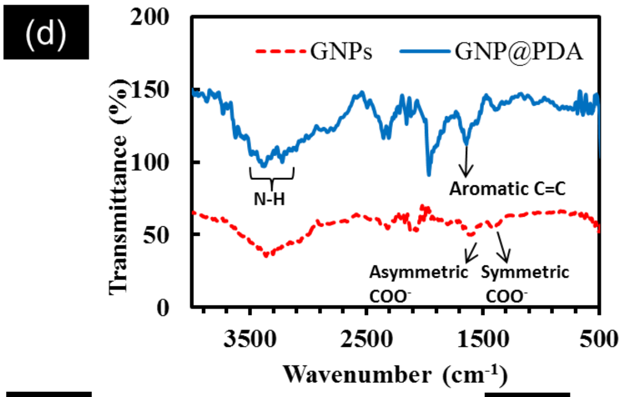

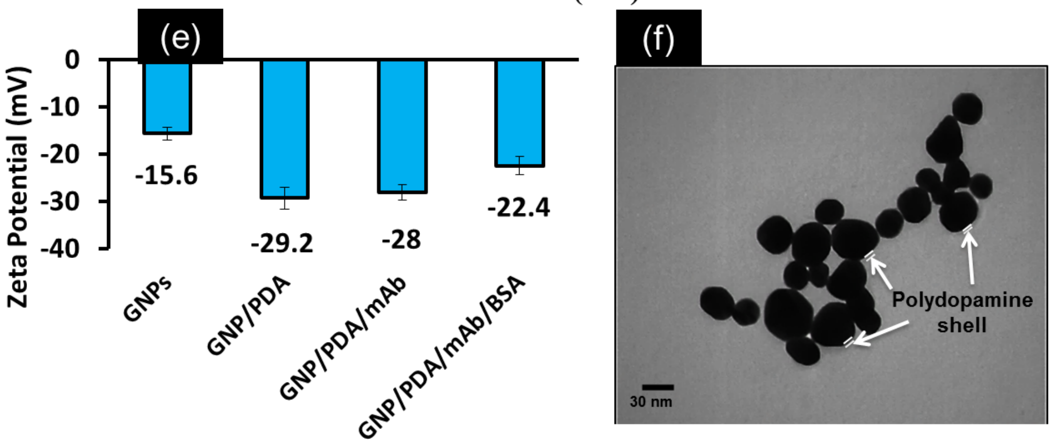

Figure S4 Characterization of nanostructures, UV-Vis spectra of different nanoparticles and respective bioconjugates **(a)** initial, **(b)** in the presence of 1% NaCl, **(c)** numerical values of λ_{max}, **(d)** FTIR-ATR spectra of GNPs and GNP@PDA, **(e)** ζ-potential values **(f)** TEM image of GNP@PDA

Table S2 Results of Tukey's multiple comparisons test on ζ-potential values

Tukey's multiple comparisons test	Mean Diff.	95% CI of diff.	Significant?	Summary	Adjusted P Value
GNP vs. GNP@PDA	13.60	8.811 to 18.39	Yes	****	< 0.0001
GNP vs. GNP@PDA-Ab	12.40	7.611 to 17.19	Yes	***	0.0002
GNP vs. GNP@PDA-Ab-BSA	6.800	2.011 to 11.59	Yes	**	0.0081
GNP@PDA vs. GNP@PDA-Ab	-1.200	-5.989 to 3.589	No	ns	0.8516
GNP@PDA vs. GNP@PDA-Ab-BSA	-6.800	-11.59 to -2.011	Yes	**	0.0081
GNP@PDA-Ab vs. GNP@PDA-Ab-BSA	-5.600	-10.39 to -0.8107	Yes	*	0.0235

Section S3:

Optimization of effective factors on the performance of the developed smartphone-based LFIA kit

In optimization experiments, running buffer spiked with CEA at two concentrations (0 as a blank and 5 or 10 ng/mL for samples containing CEA) to find the optimal values of key parameters.

Blocking of sample and conjugate pads:

The conjugation pad should be prepared so that the immuno-conjugate could be easily separated from it via lateral flow. The blocking of the conjugate pad was performed by dipping it in a solution containing 0.005 mol/L borate buffer with pH 7.5 containing 0.05% Tween-20 and 3% sucrose for 10 min followed by drying in an oven at 50 °C for 2 h. For assessment, a lateral flow test strip containing an unblocked pad was also prepared. After the addition of 2.5 µL of GNP@PDA on the conjugate pad, the strips were run by the addition of 5 µL of sample solution onto the sample pad and then adding 60 µL of running buffer (Tris buffer 0.05 M, 0.05% Tween-20). A comparison of flow behavior for two strips is shown in **Figure S5 a** and **b**. As seen, without the blocking of the conjugate pad, the immune conjugate could not waste from the pad but blocking enabled proper washing of immuno-conjugate from the conjugate pad and flow through the strip.

The impact of blocking of both conjugate pad (as described above) and sample pad with 0.005 mol/L borate buffer pH 7.5 containing 0.05% Tween-20 on flow behavior through strip was investigated in dipstick format. As seen in **Figure S5 c** and **d**, blocking of both sample and conjugate pads enables an appropriate lateral flow of conjugate along the strips. So, their blocking is necessary for the proper working of strips.

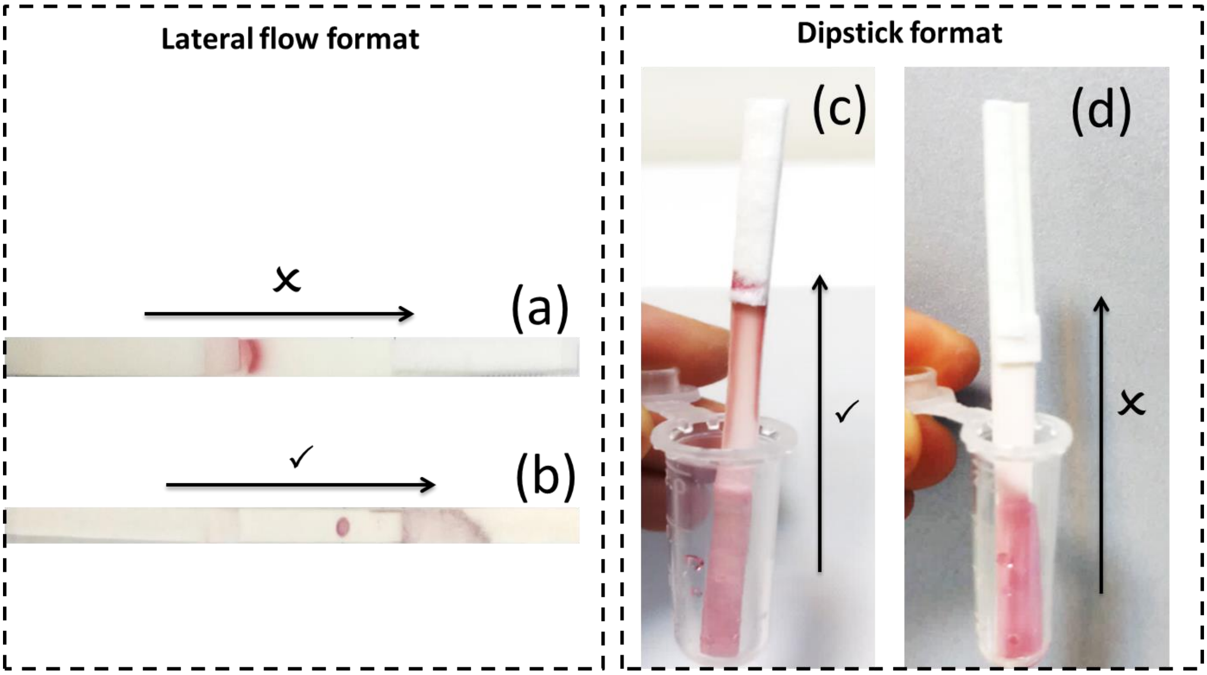

Figure S5 The flow behaviors for strips containing **(a)** unblocked conjugate pad, **(b)** blocked conjugate pad (in lateral flow format), and **(c)** both of the sample and conjugate pads have been blocked, **(d)** unblocked sample and conjugate pads (in dipstick format)

Selecting the appropriate running buffer:

The running buffer is another factor that impacts strip performance. Three common buffers were investigated including Tris (0.005 mol/L, pH 7.5), borate (0.005 mol/L, pH 7.5), phosphate (1X, pH 7.4), and all with %0.1 Tween-20. For each experiment two concentrations of CEA were examined, which 1 and 2 depict the concentration of CEA is 0 and 5 ng/mL, respectively. As seen in **Figure S6 a**, in the case of phosphate buffer (denoted as P1 and P2) the aggregation of immune-conjugate happens and the strips are not run appropriately. In addition, a considerable blank signal is observed, which hinders the suitability of this buffer for our assay. For borate buffer (denoted as B1 and B2), although the strips run well but the presence of a high background signal is clear, which is not suitable in our study. However, in the case of Tris buffer (denoted as T1 and T2), the strips run well with very low non-specific adsorption of conjugates on TZ. So, the Tris buffer was selected for

subsequent studies.

Determining the optimal value of mAb immobilized on GNP@PDA:

The amount of mAb immobilized on GNP@PDA is another important factor on assay performance. To find its optimal value, different values of mAb (1.6, 3.2, 6.4 μg of mAb for buffer samples testing and 3.2, 6.4, 9.6 μg of mAb for serum samples testing) were added drop-wise to 1 mL of GNP@PDA solution. After immobilization and blocking and re-suspending in the conjugate buffer, 2.5 μL of it was dropped on the conjugate pad and the strips were run with buffer or serum (5 ng/mL CEA). As seen in **Figure S6 b**, the values of 3.2 and 6.41 μg per mL of GNPs/PDA (denoted as b2 and s2) showed favorable behavior in the analysis of buffer and serum samples, respectively, while for lower value of mAb, very weak signals at test zone (b1 and s1), and for the higher value of mAb, the aggregation of conjugates are observed for both buffer and serum samples (b3 and s3).

Determining the optimal volume of GNP@PDA-mAb dropped on the conjugation pad:

The volume of the GNP@PDA-mAb conjugate also affects on strip performance, so different values of it including 1.5, 2.5, 5 μL (denoted as V1 to V3 depict in **Figure S6 c**) was investigated on assay performance. As seen, by increasing the conjugate volume the signal intensity increases (the right-side strips in V1 to V3 in **Figure S6 c**) but the background signal is also boosting (the left-side strips in V1 to V3 in **Figure S6 c**). In comparison, the intermediate volume of conjugate (2.5 μL) showed desired characteristics and used for the following experiments (strips denoted by V2).

Determining the optimal amount of pAb at TZ:

The concentration of pAb in the test zone also affects the performance of the assay, in which by increasing its amount, the formation of sandwich structures at test zone increases. But at high concentrations of pAb, due to the steric hindrance, it prevents the formation of the

sandwich structures. So, 0.2 µL of pAb with different concentrations (0, 0.25, 0.5 and 1 mg/mL) were immobilized on the test zone and evaluated in assay .According to **Figure S6 d**, the optimal value of pAb was 0.5 mg/mL (C3).

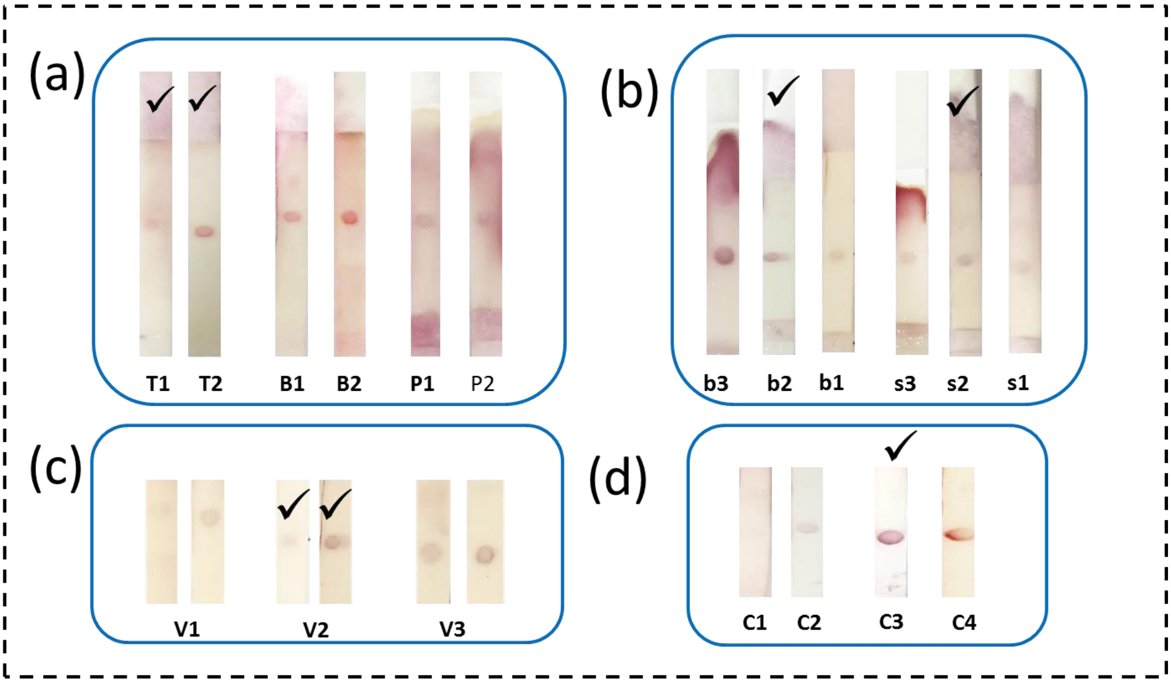

Figure S6 (a) Effect of running buffer on strip performance, T depicts Tris (0.005 mol/L, pH 7.5), B depicts borate (0.005 mol/L, pH 7.5), P depicts phosphate (1X, pH 7.4), all with %0.1 Tween-2. 1 and 2 depict the concentration of CEA is 0 and 5 ng/mL, respectively, **(b)** Effect of the amount of mAb immobilized on GNP@PDA including 1.6, 3.2, 6.4 µg for buffer samples testing (b1 to b3) and 3.2, 6.4, 9.6 µg for serum samples testing (s1 to s3) on strip performance, **(c)** Effect of the volume of GNP@PDA-mAb dropped on conjugation pad including V1 to V3 depict 1.5, 2.5, 5 µL (in each case the left image is for blank and the right image is for the sample containing CEA 5 ng/mL) on strip performance, **(d)** Effect of different amounts of polyclonal antibody immobilized on the test zone on the performance of the strip. The C1 to C4 represent concentration of 0, 0.25, 0.5 and 1 mg/mL, respectively.

References:

1. Amendola, V.; Meneghetti, M. Size evaluation of gold nanoparticles by UV− vis spectroscopy. *The Journal of Physical Chemistry C* **2009**, *113*, 4277-4285.
2. Lee, Y.B.; Shin, Y.M.; Lee, J.-h.; Jun, I.; Kang, J.K.; Park, J.-C.; Shin, H. Polydopamine-mediated immobilization of multiple bioactive molecules for the development of functional vascular graft materials. *Biomaterials* **2012**, *33*, 8343-8352.
3. Xu, L.Q.; Yang, W.J.; Neoh, K.-G.; Kang, E.-T.; Fu, G.D. Dopamine-induced reduction and functionalization of graphene oxide nanosheets. *Macromolecules* **2010**, *43*, 8336-8339.
4. Park, J.-W.; Shumaker-Parry, J.S. Structural study of citrate layers on gold nanoparticles: role of intermolecular interactions in stabilizing nanoparticles. *Journal of the American Chemical Society* **2014**, *136*, 1907-1921.
5. Liu, S.; Dou, L.; Yao, X.; Zhang, W.; Zhao, B.; Wang, Z.; Ji, Y.; Sun, J.; Xu, B.; Zhang, D. Polydopamine nanospheres as high-affinity signal tag towards lateral flow immunoassay for sensitive furazolidone detection. *Food Chemistry* **2020**, *315*, 126310.
6. Xu, S.; Zhang, G.; Fang, B.; Xiong, Q.; Duan, H.; Lai, W. Lateral Flow Immunoassay Based on Polydopamine-Coated Gold Nanoparticles for the Sensitive Detection of Zearalenone in Maize. *ACS applied materials & interfaces* **2019**, *11*, 31283-31290.
